# Challenges of safeguarding via remote consulting during the COVID-19 pandemic: a qualitative interview study

**DOI:** 10.3399/BJGP.2021.0396

**Published:** 2022-01-25

**Authors:** Sharon Dixon, Lucy Frost, Gene Feder, Sue Ziebland, Catherine Pope

**Affiliations:** Nuffield Department of Primary Care Health Sciences, University of Oxford, Oxford.; Nuffield Department of Primary Care Health Sciences, University of Oxford, Oxford.; Centre for Academic Primary Care, Bristol Medical School, Bristol.; Nuffield Department of Primary Care Health Sciences, University of Oxford, Oxford.; Nuffield Department of Primary Care Health Sciences, University of Oxford, Oxford.

**Keywords:** COVID-19, general practice, remote consultation, safeguarding

## Abstract

**Background:**

The COVID-19 pandemic required general practice to rapidly adapt to remote consultations and assessment of patients, creating new, and exacerbating existing, vulnerabilities for many patients.

**Aim:**

To explore GP perspectives and concerns about safeguarding practice during the pandemic, focusing on challenges and opportunities created by remote consultation.

**Design and setting:**

Qualitative interview study.

**Method:**

Eighteen GPs from Oxford, London, Southampton, Liverpool, Manchester, and Reading were interviewed between June and November 2020, using a flexible topic guide and fictional vignettes to explore child and adult safeguarding scenarios. Interviews were audio-recorded, thematically coded, and analysed.

**Results:**

GPs worried about missing observational information during remote consultations and that conversations might not be private or safe. Loss of continuity and pooled triage lists were seen as further weakening safeguarding opportunities. GPs experienced remote consulting as more ‘transactional’, with reduced opportunities to explore ‘other reasons’ including new safeguarding needs. However, they also recognised that remote consulting created opportunities for some vulnerable patients. While supporting known vulnerable patients was difficult, identifying new or unknown vulnerabilities was harder still. Most reported that remote consulting during COVID-19 was harder, riskier, and emotionally draining, contributing to increased GP anxiety and reduced job satisfaction.

**Conclusion:**

The GPs interviewed raised important concerns about how to identify and manage safeguarding in the context of remote consultations. Current guidance recommends face-to-face consultation for safeguarding concerns, but pressure to use remote forms of access (within or beyond the pandemic) and the fact that safeguarding needs may be unknown makes this an issue that warrants urgent attention.

## INTRODUCTION

Before the COVID-19 pandemic, most UK NHS primary care was delivered face-to-face.^1^ Evaluations of video consulting pre-pandemic had allowed doctors and patients to opt in (rather than be mandated) to use remote consultations,^2^^–^^4^ and trials recruited practices that were willing to try alternatives to face-to-face consulting. Prior to 2019, few UK practices were using video consultations.^1^^,^^5^ Evaluation of video consultations showed that they tended to be shorter than face-to-face consultations, cover fewer topics, and be less ‘information rich’.^4^ This has also been shown for telephone consultations.^6^ While offering some advantages of convenience and access, for example, for long-term condition follow-up,^7^^,^^8^ face-to-face was generally considered more appropriate for complex or highly sensitive consultations.^2^^,^^3^ Developing trust and rapport has been considered to be more difficult using remote consultations.^3^^,^^9^

The COVID-19 pandemic precipitated an almost overnight shift in consultation methods, including a move to initial contacts via telephone or email, and predominantly remote consulting.^1^ National guidance issued to GPs in England in April 2020 mandated a move towards ‘total triage’, where the initial contact required the patient to provide information about their identified need, which could then be triaged to determine the modality for the clinical consultation. This initial triage could be via telephone or electronic consultation. GPs were asked to offer telephone, video, and online consultations alongside face-to-face appointments, with advice to minimise the number of face-to-face appointments patients might require and to minimise footfall through practices to reduce infection risk.^10^

There was no evidence about how to handle safeguarding concerns in this new working environment. Safeguarding encompasses preventing harm and protecting children and vulnerable adults while providing safe and effective care.^11^^,^^12^ For GPs working in the UK, the Royal College of General Practitioners (RCGP) conceptualise safeguarding as an intrinsic part of holistic daily general practice and set out core principles including identifying vulnerability (to support and empower) and advocating for and giving a voice to those struggling to be heard.^13^ GPs are thus expected to support vulnerable patients: those with known safeguarding concerns and those with new or emergent safeguarding needs. Alongside changes in primary care, the COVID-19 pandemic response created further potential vulnerabilities, such as school closures,^14^ lockdowns, and care home isolation.^15^ There were reports of increased rates of domestic violence,^16^ impacts on young carers,^17^ reported increased rates of shaken baby syndrome^18^ and serious neurosurgical trauma to children,^19^ risks for those with learning disabilities,^20^ concerns about resuscitation decisions,^21^ and financial exploitation of vulnerable adults.^22^

**Table:** How this fits in

To the authors’ knowledge, there is no previous UK primary research exploring GP perspectives on safeguarding in primary care using remote consulting, although safeguarding has been identified as an area where face-to-face consulting is recommended. Previous research suggests that GPs find remote consulting less satisfying and less effective for those with complex needs, although safeguarding in primary care has not explicitly been studied. By focusing on GPs’ use of remote consultations in the context of safeguarding concerns in the pandemic, this study describes the losses and gains that occurred in relation to initial contact, through the consultation, and after its completion. Remote consulting adds complexity to the already challenging processes of exploring and supporting safeguarding needs in general practice.

The pandemic accelerated digital and online access, in the absence of evidence about how to manage safeguarding in this modality. Patients and the public were asked to ‘protect the NHS’, patients deemed extremely clinically vulnerable were shielding, and guidance for GPs evolved during the study period. This included early practical safeguarding guidance from the RCGP,^23^ guidance on converting virtual appointments to face-to-face if there were safeguarding concerns,^23^^,^^24^ and multi-agency guidance on managing intimate images in healthcare settings.^25^ In addition to managing patients with known safeguarding concerns, many GPs also encountered new, unanticipated safeguarding challenges in their remote encounters. This study reports GP experiences of the interface between remote consultation and the identification of potential safeguarding needs or vulnerabilities.

## METHOD

### Study design

A qualitative study was conducted using semi-structured telephone interviews. The study topic guide included fictional scenarios depicting common safeguarding situations (see Box 1 for examples and Supplementary Appendix S1 for the full topic guide and all scenarios) designed as a prompt to aid GPs to consider different safeguarding challenges when working in the pandemic. The scenarios were developed with input from safeguarding experts and were piloted with an expert advisory panel including national safeguarding leads. They were designed and used as ‘realistic’ examples of patient presentations where safeguarding issues might arise, not as exemplars. Participating GPs were advised that, *‘You will not be asked, or expected, to discuss any real cases or experiences, but your perspectives on how you approach and manage these situations, including any concerns or challenges. To support this, the researcher may introduce some fictional scenarios. They are not a test of knowledge or practice but could be used as a possible starting point for discussion and reflection.’* The interviews were conducted by one author, who is a GP and practice safeguarding lead. The wider study team and advisory group included additional safeguarding expertise.

**Box 1.** Examples of scenarios used in the interviews

A patient who you know/believe to be in a violent relationship is on your shielding list for COVID-19. You are asked to make a care plan, as her GP. A family where there is an adult with significant learning difficulties living in the family home. She usually goes to a residential day centre, but this is closed during the lockdown because of staff shortages — there is concern from neighbours about how the family are managing during the lockdown. A family where there has been previous physical chastisement with a strategy meeting last year. The family remain on a child in need plan. The father calls the practice to ask for a certificate for stress-related problems — they lost their job during the lockdown, are not sleeping, and struggling with being stuck at home (and, if asked, they have turned to drinking again). How might you approach this? What considerations does this raise?

### Sampling and recruitment

Inclusion criteria required that participants were fully qualified GPs currently working in England (with all interviews conducted during the pandemic, ensuring that all participants had relevant experience of remote consultations). Information about the study was circulated using established GP networks. With the support of the specialist safeguarding GP for that region, three clinical commissioning groups (CCGs) advertised the study with their localities (Oxfordshire, Liverpool, and North Yorkshire). Recruitment was enhanced through snowballing. The sample includes 18 GPs: 17 female and one male, from six cities (Oxford, London, Southampton, Liverpool, Manchester, and Reading), and across a range of roles (including practice safeguarding roles, locality safeguarding roles, and no formal safeguarding role). The sample included GP partners, salaried GPs, a locum, those who had qualified in the last 5 years, and others close to retirement. All worked in mainstream general practice; two held additional roles in inclusion health (working with homeless or asylum-seeking patients). The date on which the interview was conducted is included to situate them within the timeline context of the pandemic and the English societal responses and lockdowns (see Table 1 for participant characteristics).

**Table 1.** Participant characteristics

**GP number**	**GP role**	**Specialist safeguarding role?**	**Date of interview**
1	GP partner	Yes	June 2020
2	GP partner	No	June 2020
3	GP partner	Yes	June 2020
4	GP partner	Yes	June 2020
5	GP partner	No	June 2020
6	Salaried GP	Yes	July 2020
7	GP partner	Yes	July 2020
8	Salaried GP	No	July 2020
9	GP partner	No	July 2020
10	Salaried GP	Yes	July 2020
11	GP locum	No	August 2020
12	GP partner	Yes	August 2020
13	Salaried GP	No	October 2020
14	Salaried GP	Yes (inclusion health)	October 2020
15	Salaried GP	No	October 2020
16	Salaried GP	No	November 2020
17	Salaried GP	No	November 2020
18	Salaried GP	Yes (inclusion health)	December 2020

### Data collection and analysis

Telephone interviews were conducted by the lead author between June and December 2020 (interview duration 28–62 minutes). With consent, interviews were audio-recorded and transcribed verbatim. A coding framework was iteratively developed in NVivo (version 12) by three authors based on expected and emergent themes. Two authors coded the data, which were analysed thematically^26^ using mind-mapping techniques.^27^ Findings were reviewed with an advisory panel comprising five GPs, all with safeguarding roles (at practice, regional, and national levels).

## RESULTS

### Safeguarding losses and gains in the pandemic

Analysis reveals how GPs weighed up the potential losses and gains that occurred from initial contact, through the consultation, and after its completion. Findings have been grouped into the following stages: contact (gaining access to and beginning conversations with the GP); consultations; and after the consultation (the impacts of safeguarding remotely on GPs and their practice).

#### Contact (gaining access to and beginning conversations with the GP)

Several GPs described how COVID-19 had necessitated the use of shared ‘triage’ lists, where appointment and advice requests were pooled and collectively managed by doctors working that day. While remote working reduced continuity of care and made safeguarding more difficult, some GPs noted that initial remote consultations could allow them to flexibly create space for safeguarding conversations. They suggested that rapid response through pooled triage combined with the invisibility of the virtual ‘waiting room’ could encourage some conversations:

*‘A telephone call feels less to the patient like an appointment, so I think in some ways although you do have all the barrier of not having someone in front of you, it sort of facilitates sometimes going, “Oh and just while I’ve got you on the line”, or it doesn’t feel so much like they’ve got that appointment to talk about one thing. They phone the doctor to ask the doctor to call them, so I think, in some ways there might be a bit more freedom … because if you think about it, not having that list, that thing in the corner of your screen of “Two patients waiting, three patients waiting, four patients waiting”, is actually quite nice.’*

(GP11)

Working through a telephone list could allow more time for conversations with vulnerable patients, as well as the chance to offer remote or face-to-face appointments. GPs reported that this allowed them to negotiate safe times to consult with women affected by domestic violence, or to arrange a rapid assessment for adults with learning difficulties. They suggested that the possibility of a hybrid model of care, which built on the greater autonomy and flexibility facilitated by remote consulting and telephone triage, might promote a more equitable delivery of care:

*‘I think that doing more and more telephone triage you can actually clear a lot of it by phone and email which actually gives you the time to actually put to the people who need it … So actually in terms of access you are actually using for the people who need it.’*

(GP7)

However, GPs remained concerned that triage approaches that required patients to state in advance the reason for contact could deter patients and GPs from exploring any other reasons for consultation, reducing safeguarding opportunities:

*‘That’s not how consultations work. It starts off with a headache and you end up talking about alcohol and DV* [domestic violence] *, that’s the bread and butter of the conversations that we have; bullying at work or whatever the thing is, because there are things that are legitimate to go to your doctor with and there are things that really aren’t.’*

(GP3)

GPs identified some groups, such as teenagers or those with mobility needs, for whom remote consulting improved access. One GP recounted supporting a woman who was in an abusive and controlling relationship who was able to use working from home to justify locking her door for a private telephone call ‘for work’. But GPs remained concerned about vulnerable patients whose access to care was impeded by remote access. Digital exclusion due to lack of access to (or ability to use) devices or the internet, as well as barriers of language, literacy, cognitive impairment, and those with unsafe (or no) accommodation were cited:

*‘… an issue with using video is it’s dependent on, people having the right kind of phones and having data that they can use for doing video or submitting photographs, and a lot of our families don’t — you know a lot of them will have smartphones, but they don’t necessarily have the budget to use a large part of their data on video consulting.’*

(GP1)

#### Consultations

GP reflections on remote consulting conveyed their powerful sense of loss of the familiarity and boundaries of their consulting rooms. Consulting rooms were seen as *‘safe spaces’* (GP3) where GPs felt comfortable interacting with patients, and both GPs and patients welcomed the privacy and confidentiality offered. Remote consultations threatened this; GPs were concerned that they did not know who was listening in, watching, or monitoring video, online, or telephone communication. As one noted:

*‘When everyone’s locked into the house, the privacy is gone and it’s really difficult having open and honest conversations.’*

(GP10)

Some GPs attempted to mitigate this by routinely asking every contact if *‘it was safe and comfortable to speak’* (GP6). One or two noted that an advantage of not having fixed appointments was that this allowed flexibility to re-arrange calls. This was easier if vulnerabilities were known, or anticipated, but much harder to introduce unexpectedly:

*‘You ask them, “Is it OK to talk now? Are you in a private space? Can anybody hear you? Is now a good moment, would you rather I rang you back?”* […] *but I think if you’re just in a what you were thinking is a routine consultation, and you start to get little prickles it’s a little bit harder to then start to introduce that* [concerns about privacy] *. It’s harder to frame it as part of the dialogue really, suddenly to say to somebody, “Are you on your own there?” You know it’s not very subtle.’*

(GP18)

A further challenge of navigating consultations remotely was knowing how to ensure safe closure of the conversation, especially for vulnerable patients:

*‘… you want them to open up and tell you everything and then you’re kind of leaving them in their own room where they sleep and hang out.’*

(GP2)

GPs worried about what might be missed. Echoing the aforementioned concerns about access and disclosure, some reported:

*‘I think that’s where the challenge lies, because* […] *just asking isn’t necessarily going to get you the answers or uncover the problems.’*

(GP10)

*‘… that’s what worries me. The one time when I’ve really picked up horrendous domestic violence the patient rang up, her opening gambit was a physical symptom. Which was not, which was related to the violence, but the only way I realised there was violence was because I examined her. I sat her down and I said, “How did this happen?” And then she told me. But she didn’t want to tell me, the first ten minutes of the consultation she’d just focused on that physical symptom, and if I’d managed that on the phone, I would have managed it in a completely different way.’*

(GP17)

GPs wanted face-to-face visual and verbal cues and argued that *‘open communication’* (GP12) was a vital part of their safeguarding conversations. Non-verbal communication could also convey empathy, support, and *‘presence’* (GP2) in the consultation. While video consulting might restore some of these features, GPs recognised that many vulnerable people were reluctant or unable to use this technology. This sensory deprivation was felt in the context of the consultation. GPs noted that observation of the waiting room or family interactions could also inform safeguarding and care of vulnerable people, and this was also missing:

*‘You miss so much — you know concealed pregnancy, track marks on arms, poor dentition, signs of liver disease. Patients don’t phone up and say I’ve got palmar erythema and if you watch me carefully you’ll see I’m a bit trembly and you’ll see I’m drinking too much do they? So you’ll miss all of that.’*

(GP14)

*‘You don’t get the body language or the eye contact and the interaction between parents and children that you see — and not just the child that’s brought in but how the other siblings are behaving — or not — when they’re in your consulting room. That kind of information isn’t there.’*

(GP1)

One strategy GPs adopted was to use concerns about vulnerability or potential safeguarding needs as a reason to offer a face-to-face appointment. However, in the early stages of the pandemic this could be harder to negotiate, as this GP explained:

*‘Normally if I was worried about someone I would make up some sort of reason why I needed them to come and see me, like if they needed an urgent* [blood pressure] *check that I think would sound sufficiently serious and plausible that I could get them in my room so we could have a proper chat but I wouldn’t — given that we haven’t been seeing anyone besides those who we feel it is medically urgent to see I wouldn’t feel I could do that plausibly at the moment.’*

(GP5)

A particularly powerful challenge of remote consulting and adult safeguarding was when GPs needed to evaluate capacity, a task they considered virtually impossible by telephone or video:

*‘How you get someone to be their best and give their best account in the most dignified way. I’ve only ever done this face-to-face and I can’t imagine doing it in a different way.’*

(GP2)

GPs also mentioned red flags or incidents of concern, such as children who were not brought to GP or hospital appointments, who were not attending school, or where there was a delayed presentation following an injury. Such incidents were harder to appraise for relevance to safeguarding in the lockdown phases of the pandemic:

*‘We know that children were not coming in, and we were asking people not to come in, there were late presenters to A&E* [accident and emergency] […] *people fearful of the hospital and staying away, but also I guess potentially NAI* [non-accidental injury] *going missed because it’s an easy way of saying well, you know we didn’t come because we didn’t want to bother A&E during COVID.’*

(GP10)

These kinds of concerns were also more difficult to manage because of the loss of routine encounters and interactions with members of the wider primary care health team such as midwives or health visitors, or community services.

Against these largely negative concerns, GPs felt that some remote encounters created safeguarding opportunities. Video consulting allowed a view into homes of patients who would normally have ‘always’ come to the practice, and this might reveal new information about a patient’s domestic situation that could prompt further exploration. GPs also said that some patients appeared to welcome the easy access to support and advice via telephone calls. However, GPs noted that approaching safeguarding concerns in a remote consultation was even harder with patients not previously known to them. It was especially challenging to support patients in new or evolving situations, including safeguarding needs arising because of societal responses to the pandemic:

*‘Trying to demonstrate that you care which again is very hard to do on the phone particularly if you don’t know the person, I think if you do know that patient and you already have a relationship with them you might stand a chance on the phone.’*

(GP5)

GPs worried about vulnerable adults and children losing regular contact with family members, losing access to respite care, day centres, and schools, increased alcohol consumption, increased domestic violence, and impacts on young carers, including those newly created by COVID-19. Yet they also felt that the pandemic had created opportunities for proactive care and support for patients. GPs described working with social prescribers and using the shielding programme as a valid reason to contact vulnerable patients. They also proactively established networks and groups to support new families. Some aspects of multi-agency communication for safeguarding, such as liaising with community health workers, health visitors, and social care, also evolved rapidly during the pandemic and was welcomed. Working remotely removed the necessity to travel and made it easier to join safeguarding meetings and case conferences, as explained here:

*‘I have taken part of a strategy meeting that I perhaps otherwise wouldn’t have been able to* […] *I could attend for the amount of time that I could spare, whereas previously I would have said that I can’t attend but because I was doing it remotely from my consulting room, I did it for the length of time that I was able to allocate. So, I think actually, that was a good change in practice.’*

(GP1)

**Figure 1.**
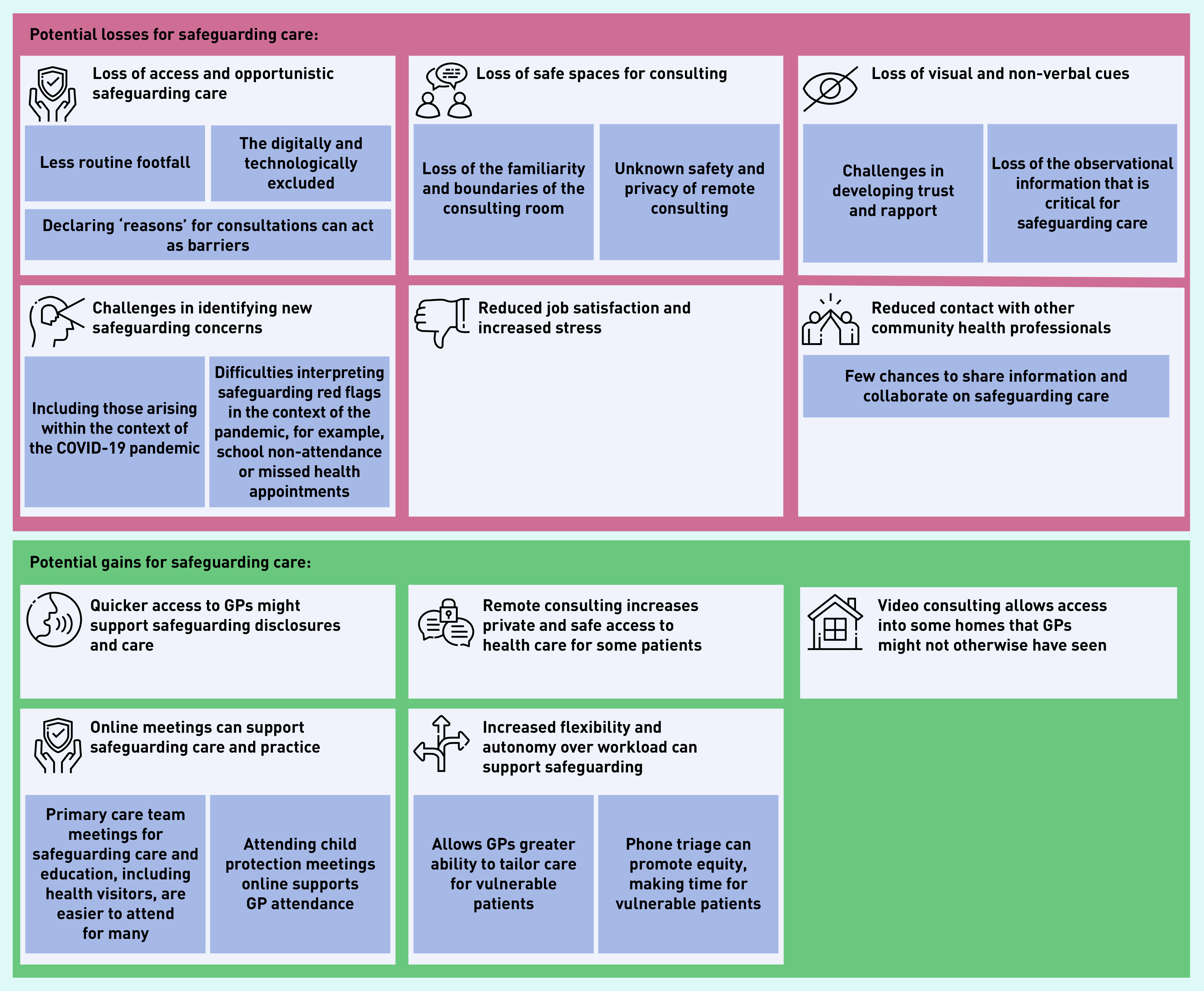
*Balance of losses and gains for safeguarding utilising remote consultations during the COVID-19 pandemic.*

Figure 1 summarises the gains and losses for safeguarding experienced by interviewees.

#### After the consultation (the impacts of safeguarding remotely on GPs and their practice)

GPs described emotional and personal impacts of the transition to remote assessment and consultations. While these were brought to the fore in complex areas of their jobs, including safeguarding and end-of-life care, they were apparent in other consultations. GPs reported a range of potential stressors, including worrying about missed or delayed diagnoses and missed opportunities for being physically present with patients at pivotal moments of care, for example, conveying a life-changing diagnosis or sharing a positive event such as a patient being granted asylum status. For some GPs, their role had become stressful and *‘lonely’* (GP8), *‘isolated’* (GP13), or *‘anxiety provoking’* (GP17), resulting in *‘more sleepless nights’* (GP16) and worry. Remote working was *‘less rewarding’* (GP5) and concerns were raised about a lost ability to make an *‘intuitive’* (GP9) assessment of situations, with potential impacts for training and learning in primary care. The net effect was that primary care had become more *‘transactional’* (GP18), and what was enjoyable and valuable within GPs’ roles was reduced:

*‘I enjoy seeing patients, losing that face-to-face impact interaction, I think has made a massive impact. To be honest, I don’t really enjoy the job at the moment.’*

(GP11)

*‘I feel as a doctor that quite a lot of the joy and the reason why I still want to get up and go to work as a GP has been taken away from me, because of the fact that I’m not doing much face-to-face stuff now. And I just rely so much, I’m so much better face-to-face, than on the phone.’*

(GP12)

*‘A big part of what we do as GPs is risk assessment* […] *So you are making risk assessments in situations where you don’t have as much experience, and that I think has been a source of stress. And particularly for potential high-risk situations like safeguarding. Those are stressful ones anyway without the added challenges of remote consulting.’*

(GP1)

Critically, some, including both newly qualified and more experienced GPs, reported remote consulting was changing how they felt about staying in general practice:

*‘Right at the beginning of all this COVID crisis — I became the doctor I needed to be but it wasn’t the doctor I wanted to be* […] *I want to go back to being the doctor I want to be. And not lose some of the good stuff but I don’t think the doctor I want to be is on video, that might be me being set in my ways. That would be my personal challenge not to lose my emotional connection with patients.’*

(GP2)

**Figure 2.**
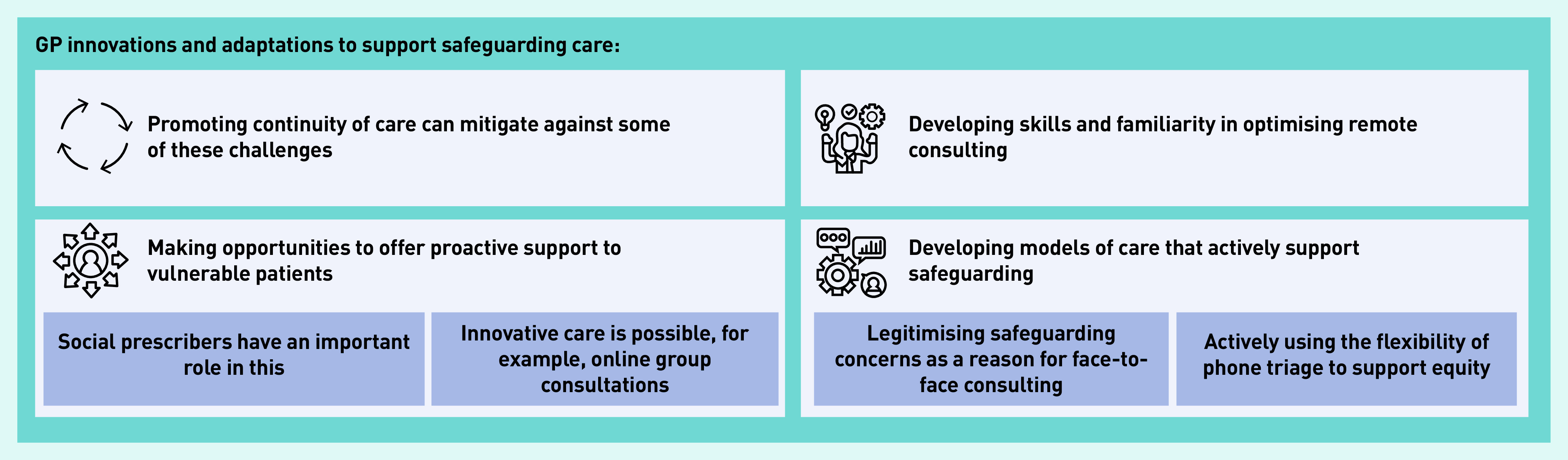
*GP innovations and adaptations to support safeguarding care.*

To address these kinds of challenges a number of GPs told of how they had made positive changes to adapt to remote working, including innovative and proactive group consulting, developing team meetings and peer support, and of personal skillset development to optimise remote consulting. These are summarised in Figure 2.

## DISCUSSION

### Summary

Remote consulting adds complexity to the already challenging processes of safeguarding in general practice. This study articulates GP perspectives on safeguarding during the pandemic. GPs identified concerns throughout the patient journey, from impacts on access and accessibility, to navigating safety, privacy, and confidentiality while consulting, and barriers to disclosure within the consultation (including impacts on trust and rapport, and how difficult it was to create space for patients to bring up other concerns). The loss of visual cues and truncated communication were pervasive concerns. Following the consultation, the changes to wider team engagement and communication brought both challenges and opportunities. GPs described ways that they were responding to these challenges and they identified opportunities for innovative consultation approaches to support potentially vulnerable patients. However, they also revealed concerns about the impact of remote working on their stress and job satisfaction, which could impact on future primary care recruitment and retention.

### Strengths and limitations

This study adds GP perspectives to the emerging picture of the impact of COVID-19 on UK general practice and where and how remote consulting should sit within primary care post-pandemic. This study adds nuance and depth to current understanding of remote consulting in the context of safeguarding needs. Participating GPs were from a range of primary care roles, including those with and without specialist safeguarding roles. The analysis was supported by an expert advisory panel of GPs who confirmed the credibility of the themes identified. Qualitative interviews were limited to GPs who chose to participate and who were interested in safeguarding; therefore, the authors cannot (and do not) suggest that all perspectives have been heard. However, the accounts gathered suggest a range of perspectives have been captured, and the analysis adds to a field where there is scant evidence. The study does not include patients’ perspectives or experiences of remote consultations that concerned safeguarding issues during the pandemic and these accounts are needed.

### Comparison with existing literature

The authors did not identify any primary research exploring GP perspectives on safeguarding in primary care using remote consulting. Safeguarding has been identified as an area where alternatives to remote consulting should be considered.^28^^,^^29^ The analysis presented here further justifies this guidance and highlights specific concerns of GPs, as well as their strategies mitigating against these challenges. In August 2020, mid-way through interviews and data collection, guidance was issued advising GPs to *‘Remain professionally curious and vigilant. Consider safeguarding issues and whether you can explore these fully via a remote consultation. If you have safeguarding concerns at any stage, you should convert a remote consultation to a face-to-face assessment, unless there are compelling reasons why that cannot happen* . *’* ^29^ The findings presented in this study support this guidance and emphasise its importance in policy.

Murphy *et al* ’s mixed-methods study, exploring primary care’s transition to remote consulting, also identified that GPs found it less satisfying and effective for those with complex needs, although safeguarding was not explicitly mentioned.^1^ GPs reported reduced enjoyment and challenges related to the lack of verbal cues, contributing to a sense of cumulative fatigue and holding more risk.^1^ As in the present study, GPs valued the greater flexibility and control offered by remote consulting. In their review of evidence for remote social work delivery of adult safeguarding, Anka and colleagues also raised concerns that it can be harder to develop trust and rapport, and to identify abuse.^30^ They identified that remote capacity assessment was problematic, as did participants in the present study who judged that these assessments should almost never be conducted remotely. As with pre-pandemic evaluations,^3^ present study participants identified difficulties developing new relationships and establishing trust when working remotely. That it is harder to identify ‘other’ concerns on the telephone has been documented by previous research.^6^

When considering the impacts of remote assessment and digital encounters on access to care, the Association for Young People’s Health (AYPH) sought the views of young, marginalised groups. Like the GPs interviewed in the present study, AYPH documented concerns about the impact of the pandemic on young carers.^31^ While digital routes could help some young people access care, there were concerns about the barriers for those without access,^31^ which corresponds with present study findings. A mixed-methods evaluation assessing the impact of total triage and remote consulting on vulnerable patient groups^32^ adds patient voices to the concerns raised by GPs in the present study, showing, for example, that patients may be reluctant to tell reception staff the reason they want to see a GP or that they may not be able to afford internet or smartphone charges incurred for remote consultations. GPs in the present study felt that they were not as able to identify safeguarding concerns during the pandemic. This is critical to consider in the context of reports of markedly reduced child safeguarding referrals during the pandemic, despite concerns that the risk of abuse may be higher.^33^ The reasons why referrals reduced needs further exploration.

### Implications for research and practice

Even as the regulations introduced into primary care in the context of the pandemic wane, rates of remote consulting remain significantly higher than they were pre-pandemic, and remote consultations are expected (alongside face-to-face consultations) to remain an important part of delivering primary care in England.^34^ While there has been guidance^35^ and policy directives^36^ to increase face-to-face consultations, the 2021–2022 Operational Guidance published for the NHS in England also calls for primary care systems to significantly increase online consultations and to embed total triage.^37^ This study highlights the potential relevance of both triage and consultation modality to the processes of primary care safeguarding. Therefore, the findings of this study remain highly relevant and timely for consideration in policy and practice as primary care in England re-formulates as it emerges from the pandemic, where it is envisaged that the modality of consultation will be determined by a process of shared decision making between practitioner and patient.^34^

Safeguarding is a core, but often overlooked, part of holistic general practice. Changes to consulting and primary care, necessarily introduced during the COVID-19 pandemic, complicate this already difficult role. Considering (and asking about) timing, location, and safety should be part of every remote consultation. Recognising the limitations of remote consultation (including loss of visual cues and the potential barriers to disclosure) and converting from remote to face-to-face appointments, where there is uncertainty or any potential concerns about safeguarding needs or vulnerability, is now part of national guidance and is supported by the evidence presented in this study *.* While this can be planned if safeguarding needs are known or anticipated, all clinicians working remotely need to be aware of the possibility of unexpected needs arising. Primary care teams and policymakers need to be aware of the potential impacts of asking patients to declare reasons for consultation, whether this is by telephone or online consulting, and retain their professional curiosity about ‘other’ concerns. Erosions or reductions in continuity of care can have impacts on safeguarding opportunities and effectiveness — practices and practitioners could hold this awareness as they develop their practice systems. Challenges to access may be mitigated and working flexibly can create space for equity and time for those in need, and the authors suggest this could be actively nurtured. However, there are real perils in missed observations and reduced opportunities for safe disclosure; essential steps towards safeguarding care include multi-agency referral and collaboration.

The impacts of remote consulting on how GPs manage safeguarding merits further attention as primary care comes out of the pandemic. This includes urgent consideration of the impacts on GPs’ professional satisfaction and emotional wellbeing.
